# The Discrepancies of COVID-19 Clinical Spectrum Between Infancy and Adolescence – Two Case Reports and a Review of the Literature

**DOI:** 10.3389/fped.2020.577174

**Published:** 2020-10-30

**Authors:** Cristina Oana Mǎrginean, Lorena Elena Meliţ, Maria Oana Sǎsǎran

**Affiliations:** ^1^Department of Pediatrics I, George Emil Palade University of Medicine, Pharmacy, Science, and Technology of Târgu Mureş, Târgu Mures, Romania; ^2^Department of Pediatrics III, George Emil Palade University of Medicine, Pharmacy, Science, and Technology of Târgu Mureş, Târgu Mures, Romania

**Keywords:** COVID-19 infection, children, lack of symptoms, atypical cases, peculiarities

## Abstract

A new pandemic caused by SARS-CoV-2 raised new challenges for the worldwide healthcare system, involving the pediatric field since children own certain peculiarities that caused a different reaction to this infection as compared to adults. We report two cases of COVID-19 in two pediatric patients, a 6-month-old male infant and a 15-year-old female teenager in order to underline the age-related differences in terms of clinical manifestations. Thus, the 6-month-old male infant was admitted in our clinic presenting fever, rhinorrhea and diarrhea for ~24 h. Taking into account that both parents presented respiratory manifestations, nasopharyngeal/oropharyngeal swab-based polymerase chain reaction tests for SARS-CoV-2 were performed, and the test came back positive for the parents and inconclusive for the infant. Nevertheless, the infection was confirmed also in the child by the second test. The symptoms resolved in the 2nd day of admission with symptomatic treatment. The 2nd case, a 15-year-old female teenager, presented to the emergency department with fever, cough and shortness of breath (O_2_ saturation 84%). The chest radiography pointed out multilobar impairment. The nasopharyngeal/oropharyngeal swab-based polymerase chain reaction test for SARS-CoV-2 infection was positive. She was admitted to the intensive care unit for 3 days, and the evolution was favorable with anti-viral therapy. The pediatrician's awareness regarding both asymptomatic and atypical cases is vital for decreasing the transmission of this novel life-threatening condition.

## Introduction

In December 2019, a new type of coronavirus emerged in the province Wuhan, China, which expressed the ability of fast spreading worldwide leading to a real pandemic since March 2020 according to the World Health Organization. On 11th of February, this virus was named “Severe Acute Respiratory Syndrome Coronavirus 2 (SARS-CoV-2),” and the disease caused by it is known as coronavirus disease-19 (COVID-19). Recent research has proved that SARS-CoV-2 belongs to genus β, a new coronavirus family, originating from bats, and it enters cells by binding to the angiotensin converting enzyme 2 (ACE-2) receptor (1). Over this last 6 months, millions of people worldwide were affected by this novel disease resulting in more than 400,000 deaths. COVID-19 affected predominantly adults, and the number of children confirmed with this infection reached only 0.02%, most of them presenting a history of family exposure or contact with infected adults, but a recent travel in affected areas was also described in rare cases (1). The first case of SARS-CoV-2 infection described in a pediatric patient was reported on January 20 in Shenzhen (2). Taking into account this discrepancy in terms of age-related differences, many researchers tried to find the causes of this phenomenon postulating different hypotheses. Thus, the immaturity of immune system and subsequent immune responses might contribute to the a lower susceptibility to this infection in children as compared to adults (3). Another explanation might consist of the improper distribution, maturation and functioning of ACE-2, an essential viral receptor for SARS-CoV-2 (4). Moreover, viral co-infection might play a key role in the limited replication of SARS-CoV-2 due to virus-to-virus interaction and competition (5). The protective role of Bacillus Calmette-Guérin vaccine was also postulated as a possible theory in order to explain the lower incidence of COVID-19 in pediatric patients since it is well-documented that this type of vaccine might result in heterologous immunity to other pathogens. This phenomenon is known as “trained immunity” and it involved innate cells, among which monocytes, macrophages and epithelia (6). In spite of the high relevance of all these theories, the interaction between these peculiarities encountered in children is the most-likely explanation for the discrepancies between age groups in term of COVID-19.

The clinical spectrum of SARS-CoV-2 in children is difficult to be delineated at this time due to the scarce literature reports in this age group. Moreover, most of the children confirmed with this infection are asymptomatic according to Dong et al. who showed in a large pediatric review involving 2,143 cases that 13% of those virologically confirmed presented no symptoms (7). This fact might represent a real epidemiological burden since these asymptomatic children are less likely to be tested, but unfortunately, they contribute to the transmission of this virus. Thus, reports from Wuhan in terms of clinical characteristics of children diagnosed with COVID-19 underlined that out of 171 children, 15.8% were asymptomatic carriers and a single patient required ventilator support (8). Additionally, a study that compared Chinese children with COVID-19 and with severe acute respiratory syndrome 2003 (SARS) revealed that none of the patients with SARS was asymptomatic as compared to 29.1% of the COVID-19 patients who were asymptomatic on admission and 20.9% expressed no symptoms throughout their hospitalization (9). The authors found that fever was present in almost all patients with SARS as compared to 51.3% of COVID-19 patients, and oxygen supplementation was required frequently in SARS patients, i.e., 18.6 vs. 4.7% of COVID-19 children.

Besides the above stated benefit of the commonly encountered viral co-infection in children, it might be an important disadvantage in terms of SARS-CoV-2 detection (10). Moreover, usually those that present symptoms express a mild form of COVID-19 disease with a good prognosis, severe pediatric infections being reported seemingly rare (11). Thus, only 2.5% of children reported by The February 2020 World Health Organization-China Joint Mission on Coronavirus disease developed severe disease in comparison to 13.8% overall, and critical forms were diagnosed in 0.2% of pediatric patients as compared to 6.1% overall (12). Nevertheless, among the most common clinical symptoms expressed by children with SARS-CoV-2 were those reported also in the context of influenza, such as fever, cough, sneezing, a sore throat, fatigue, and myalgia (7). A thorough anamnesis based on the clinical skills of the pediatrician might reveal an important epidemiologic risk factor increasing the diagnosing rate (13). The diagnosis of SARS-CoV-2 in children is definitely a real challenge for physicians and the increased awareness is the single factor that might contribute to the improvement of diagnosing rate in this age group. Thus, a proper description of the clinical spectrum of this condition in children is most-likely the key factor in terms of diagnosis since clinicians conceive their management and investigations based on patient's symptoms.

We report two cases of COVID-19 in the extremes of pediatric ages, a 7-month-old infant and a 15-year-old female teenager, in order to underline the age-related wide diversity of symptoms.

*The written informed consent was obtained from both patients' mothers prior to the publication of these case reports*.

## Case Reports

### Case 1

#### Presenting Concerns

A 6-month-old male infant was admitted in our clinic for fever (38.3°Celsius), rhinorrhea and diarrhea. The onset of the symptoms was ~24 h before admission. We must mention that both parents presented respiratory symptoms. The patient's history revealed that he was obtained by *in vitro* fertilization, fully vaccinated according to the national recommendations, with appropriate development for his age, and no pathologies up to this age.

#### Clinical Findings

The clinical exam revealed influenced general status, serous rhinorrhea, pharyngeal erythema and diarrhea (~5 stools/day). The patient weighed 7,700 g.

#### Diagnostic Focus and Assessment

The laboratory tests performed on the day of admission pointed out microcytic anemia (hemoglobin 10.7 g/dL, medium erythrocyte volume 75.9 fL), low erythrocyte count (3.99 × 10^6^/μL), neutropenia (1,070/μL), a normal count of leukocytes (8,250/μL) and lymphocytes (2,540/μL), and a C-reactive protein (CRP) of 2.2 mg/L. The tests for other viral infections (Influenza A and B, Syncytial virus) were negative. Taking into account the epidemiological context, nasopharyngeal/oropharyngeal swab-based polymerase chain reaction tests for SARS-CoV-2 infection were performed for the infant and his parents. The test was positive for both parents, but in the infant the result was inconclusive. Thus, we repeated the test and the second test confirmed the infection in our patient too. No signs of pediatric multisystem inflammatory syndrome (PIMS-TS) or organ failure were identified during the clinical course of this case.

#### Therapeutic Focus and Assessment

After the admission we initiated symptomatic treatment with antipyretics in case of fever and anti-diarrheic drugs (diosmectite and racecadotril). The fever persisted only for 1 day as well as diarrhea. Thus, when the second test for SARS-CoV-2 infection came back positive, the infant was asymptomatic, and the chest radiography revealed no pathological findings.

#### Follow-Up and Outcome

After ~4 days of hospitalization in our clinic, the patient was transferred to the infectious diseases clinic taking into account that his mother was admitted there, but he was asymptomatic and his clinical course was outstandingly favorable. No sequelae or unanticipated events as a part of the patient's follow-up.

### Case 2

#### Presenting Concerns

A 15-year-old female teenager presented to the emergency department with fever, cough and shortness of breath. Her family history revealed that both parents were positive for SARS-CoV-2 infection. The personal history underlined no important pathologies up to this age, and the vaccines were administered according to her age and the national recommendations.

#### Clinical Findings

The clinical exam at the time of admission showed influenced general status, fever (39°Celsius), dry cough, and an O_2_ saturation of 84%. The patient weighed 46 kg.

#### Diagnostic Focus and Assessment

**Figure 1:**
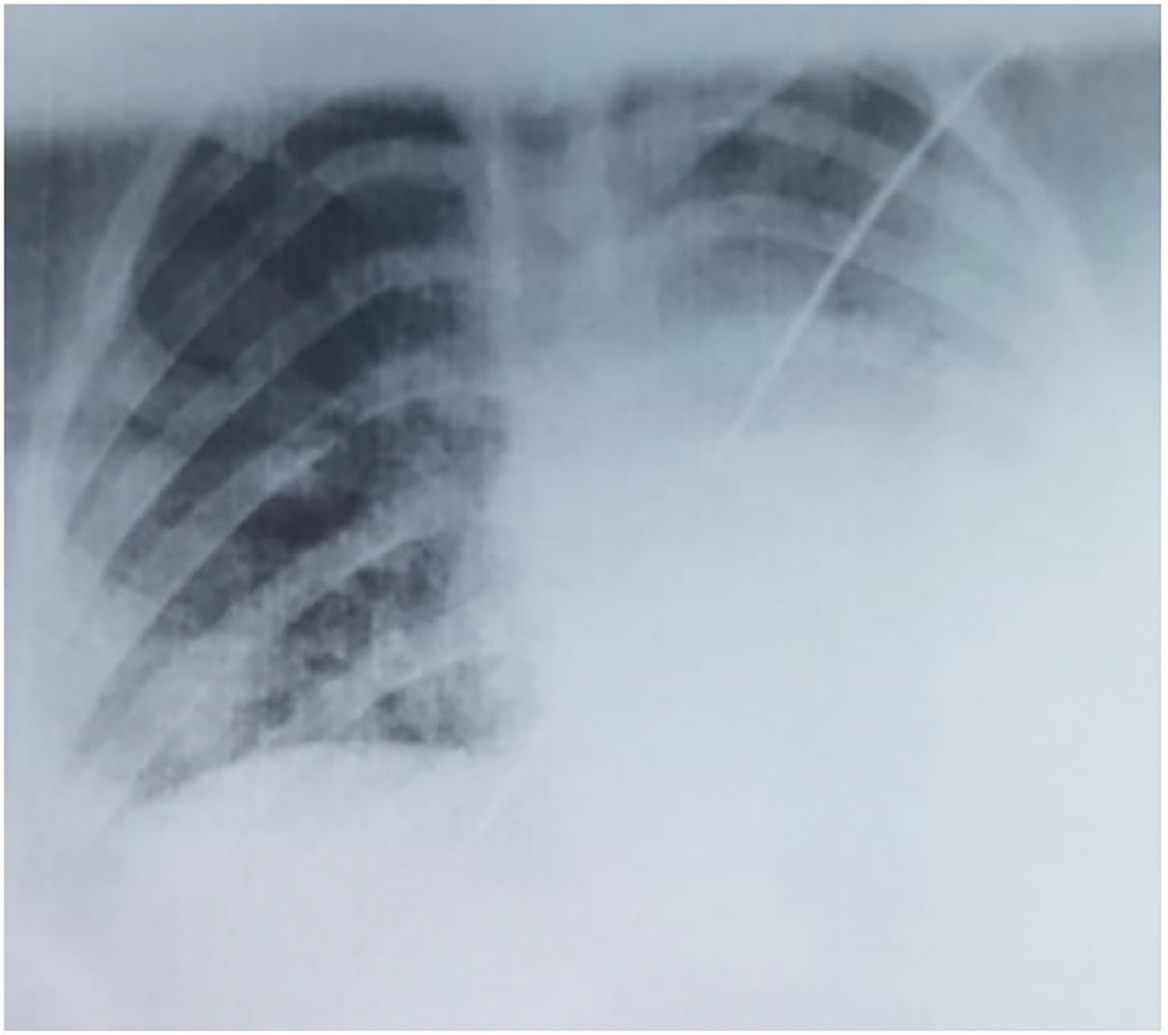
Radiological aspect of SARS-Cov-2 pneumonia in our case.

The laboratory tests pointed out lymphopenia (940/μL), thrombocytopenia (136,000/μL), a neutrophile count within the normal ranges (3,010/μL), a normal leukocyte count (7,910/μL), elevated lactate dehydrogenase (776 U/L), and a CRP of 4.1 mg/L. The tests for other viral infections (Influenza A and B) were negative. The thoracic radiography showed multiple asymmetric patchy infiltrates within both lungs, certain consolidations presenting a surrounding halo (Figure 1), with a similar aspect on the chest CT. Taking into account her history, clinical exam, laboratory parameters and radiological findings, we performed a nasopharyngeal/oropharyngeal swab-based polymerase chain reaction test for SARS-CoV-2 infection, which confirmed our diagnosis of COVID-19. The patient presented no signs of PIMS-TS and no signs of organ failure were identified during her clinical course (Table 1).

**Table 1:** The laboratory parameters of the two patients.

	**Leu (X 10^**3**^/μL)**	**Lymph (X 10^**3**^/μL)**	**Neu (X 10^**3**^/μL)**	**Plt (X 10^**3**^/μL)**	**Ery (X 10^**6**^/μL)**	**Hb (g/dL)**	**MCV (fL)**	**CRP (mg/L)**
Infant	8.25	2.540	1.070	256	3.99	10.7	75.9	2.2
Adolescent	7.91	0.94	3.01	136	4.72	12.4	81.6	4.1

*Leu, leucocyte; Lymph, lymphocyte; Neu, neutrophils; Plt, platelets; Ery, Erythrocyte; Hb, hemoglobin; MCV, mean corpuscular volume; CRP, C-reactive protein*.

#### Therapeutic Focus and Assessment

She was admitted to the intensive care unit for 3 days requiring intubation during the first 24 h with favorable evolution after the initiation of antiviral therapy (lopinavir/ritonavir in a dose of 200/50 mg twice a day) associated with mask O_2_ supplementation, antibiotics (ceftriaxone in a dose of 1.5 g twice a day), antipyretics (paracetamol) and analgesics (ibuprofen 200 mg three times a day).

#### Follow-Up and Outcome

After 3 days of hospitalization in the intensive care unit, the patient was further referred to the infectious diseases clinic for another 5 days where the antiviral and antibiotic therapy was continued, presenting favorable evolution and no sequelae were further described. Fortunately, no side-effects were noticed as a result of antiviral therapy and the patient presented a good tolerability.

## Discussions

Children represent a particular age group in the era of COVID-19 pandemics since they express a lower incidence, milder forms and a better prognosis as compared to adults. Thus, a large review from China comprising 72,314 subjects, of which 44,672 were found positive for COVID-19, underlined that only 2% were aged between 0 and 19 years (14). Other countries worldwide reported similar incidences of pediatric COVID-19, ranging between 1 and 5%, with an even lower hospitalization rate (15, 16).

The symptoms in children confirmed with SARS-CoV-2 infection were reported to be less severe as compared to adults or they might be even absent (7). The most common symptoms reported by Dong et al. after assessing 2,143 children, of which 34.1% were confirmed with COVID-19 and the remainder were considered to have clinically suspected disease, were fever, cough, sneezing, a sore throat, myalgia, fatigue and wheezing (7). Another study that included Chinese children noticed that cough was present in 48.5% of the cases, pharyngeal erythema in 46.2%, while fever of at least 37.5°C was reported in 41.5% of the patients (8). The temperature value reported by different authors that assessed children with COVID-19 usually varies between 38.1 and 39°C (8, 17). Both our cases presented fever above 38°C, with a higher value in case of our older patient. Nevertheless, pharyngeal erythema was present only in our infant, while cough and shortness of breath were expressed by the female teenager described above. Moreover, our older patient presented an oxygen saturation of 84%, which is a sign of severe disease along with dyspnea and central cyanosis (7). Gastrointestinal symptoms were also described to be part of the clinical spectrum of COVID-19 patients. Thus, data from China proved that up to 79% of the patients diagnosed with this condition complained of diarrhea, loss of appetite, nausea, vomiting, gastrointestinal bleeding and abdominal pain (18). Assessing the data from the literature regarding gastrointestinal symptoms in COVID-19 patients, Tian et al. noticed that loss of appetite was the most common symptom in adults accounting for up to 50.2% of the cases, whereas diarrhea was frequently encountered in both adults and children ranging between 2 and 49.5% of the patients (19). A valuable report from Wuhan which compared 244 COVID-19 children with or without gastrointestinal symptoms showed that 34 of them complained of at least one gastrointestinal symptom (diarrhea, nausea, vomiting, abdominal pain, loss of appetite) on admission, more than half being under 3 years of age (20). Similarly, in our cases, the presence of gastrointestinal symptoms was identified in a 6-month-old infant. As a result of multivariate analysis, the authors proved that age and fever were significant predictive factors for the presence of gastrointestinal symptoms on admission (20). Besides his small age, our infant also presented fever on admission emphasizing the findings of Xiong et al. Another study that assessed 31 pediatric patients with COVID-19 noticed that three of them presented diarrhea as the first symptom without fever and cough (21). Thus, diarrhea might be an initial symptom of patients with this novel condition, but it can also occur after the onset or as a result of anti-viral therapy. In the study of Fang et al. out of 295 patients, 146 had diarrhea, but in only 22.2% of them this symptom was present before diagnosis (18). The duration of this symptom varied between 1 and 14 days, with a mean of 4.1 ± 2.5 days, while the frequency was 3.3 ± 1.6 per day with yellow-watery stools in 34.3% of the cases (18). The treatment for these cases consisted only of symptomatic treatment and rehydration therapy when needed (22). Another study that included 204 cases of COVID-19 patients underlined the presence of gastrointestinal symptoms in almost a half of the patients and seven cases expressed only gastrointestinal symptoms without respiratory manifestations (23). Moreover, it was underlined that the patients with digestive symptoms tend to have a worse prognosis as compared to those without these features with a discharge rate of 34.3 vs. 60% (19). Our infant also presented diarrhea before diagnosis associated with fever, but without important respiratory symptoms, except for a runny nose. Similar to the previously mentioned data, the symptoms lasted ~2 days and they resolved with symptomatic treatment. Contrariwise, the older patient described above had no digestive manifestations, but the respiratory symptoms marked the course and severity of the disease. Taking into account the presence of digestive symptoms in patients with COVID-19, a fecal-oral transmission of this novel virus was hypothesized. Thus, according to Doremalen et al. the virus is viable in aerosols for at least 3 h, whereas, on plastic and stainless-steel surfaces it may survive up to 3 days (24). Ong et al. performed a study on patients with confirmed fecal positivity for SARS-CoV-2 by PCR without diarrhea and they noticed that the samples from the door handle, the surface of the toilet bowl, and inside bowl of the sink were positive (25). Further studies emphasized the presence of time window in terms of positivity from different tissue specimens (26, 27). Thus, it was proved that fecal nucleic acid became positive at 2–5 days after the specimens from the respiratory system, and in up to 82% of the patients the fecal tests remained positive after their respiratory specimens became negative (26, 27). These findings definitely suggest that virus particles survive longer in the gastrointestinal tract when compared to the respiratory one. Moreover, the virus might spread easily *via* feces in case of an increased viral load in the stools or a virus-friendly environment (19). Fecal-mucosal or fecal-aerosol-respiratory transmissions are also possible in case of exposure to public toilets, areas with poor sanitation or between family members by sharing the same toilet (19). Similarly, Chen et al. performed a study on 42 patients with SARS-CoV-2, and found that 28 tested positive in stool specimens, while only 8 had gastrointestinal symptoms, suggesting that the presence of SARS-CoV-2 in feces is not related to either the presence of gastrointestinal symptoms nor the severity of this novel condition (28). Pediatric patients were proven to follow the same pattern according to another study that assessed the nasopharyngeal and rectal swabs of 10 children and proved the persistence of positive rectal swabs in 8 of them even after viral clearance from the nasopharyngeal specimens (29). Unfortunately, we could not perform a PCR of the stool specimens in our cases since this was not available in our hospital, but most-likely in would have been positive at least in the infant.

Fortunately, the prevalence of severe and critical forms is low in children as underlined in a large review: 10.6% below the age of 1 year, 7.3% in those between 1 and 5 years of age, 4.2% between 6 and 10 years, 4.1% between 11 and 15 years, and 3% between 16-17 years (7). The same authors reported that half of the pediatric patients who developed critical forms of COVID-19 were below the age of 1 year (7). These data suggest that the prevalence of severe and critical forms is inversely related to age underlining that infants and pre-school aged children might express an increased vulnerability for COVID-19 morbidity similar to other infections (30). These age subgroups should represent a concern for the clinicians together with other subgroups at increased risk, like those with pulmonary underlying disease or other immunocompromising conditions (31). Contrariwise, the infant presented above had a milder form and a considerable shorter evolution as compared to the teenager. The first case of an infant diagnosed with COVID-19 was reported by Zhang et al. describing a 3-month-old female whose evolution was similar to our case presenting fever for 1 day, but the thoracic radiography pointed out opacities within both lungs (23). Nevertheless, the chest radiography in our infant showed no pathological findings as compared to the older patient, in whom we noticed similar findings to those reported in the literature with multilobar involvement (32). In terms of laboratory parameters, a review that included 66 children pointed out normal leucocyte count in 69.2% of the cases, neutropenia in 6%, neutrophilia in 4.6% and lymphopenia in 3%, while C-reactive protein was elevated only in 13.6% of these children (11). In our cases, the infant presented neutropenia, while the teenager patient was found with lymphopenia, both being encountered with a leucocyte count within the normal ranges and normal CRP level.

It is true that much has been learnt about this novel life-threatening condition in a very short time, but in case of children there is still much to be clarified in order to properly characterize COVID-19 in this age group. An accurate characterization of this condition requires time, and each pediatric case diagnosed with this still novel coronavirus infection should be described and reported in the literature for the completion and coverage of all aspects regarding this disease.

The limitations of this case series consist mainly in the fact that we reported only two cases diagnosed with COVID-19, but also that the viral load could not be assessed in our patients. Moreover, the assessment of stool specimens would have been useful, but unfortunately, it was not available in our hospital since these cases were diagnosed in the early period of this pandemic. Nevertheless, we reported two pediatric patients with COVID-19 underlining the huge discrepancies between their clinical spectrum and evolution most-likely due to the difference in their ages. Taking into account the limited data on pediatric patients with COVID-19, our case reports would definitely add valuable information to the clinical practice and not only to the scientific field.

## Conclusions

The wide-spectrum of the clinical manifestations encountered in children with COVID-19 is a real challenge for establishing an early diagnosis. Both respiratory and digestive symptoms define the infection with SARS-CoV-2 in pediatric patients. The pediatrician's awareness regarding both asymptomatic and atypical cases is vital for decreasing the transmission of this novel life-threatening condition.

## Data Availability Statement

The raw data supporting the conclusions of this article will be made available by the authors, without undue reservation.

## Ethics Statement

Written informed consent was obtained from both patients' mothers prior to the publication of these case reports.

## Author Contributions

CM and LM conceptualized, designed the study, drafted the initial manuscript, and reviewed and revised the manuscript. MS designed the data collection instruments, collected data, carried out the initial analyses, and reviewed and revised the manuscript. All authors approved the final manuscript as submitted and agree to be accountable for all aspects of the work.

## Conflict of Interest

The authors declare that the research was conducted in the absence of any commercial or financial relationships that could be construed as a potential conflict of interest.
